# Role of Microbiome in Modulating Immune Responses in Cancer

**DOI:** 10.1155/2019/4107917

**Published:** 2019-06-12

**Authors:** Mukulika Bose, Pinku Mukherjee

**Affiliations:** Department of Biological Sciences, University of North Carolina at Charlotte, Charlotte 28223, USA

## Abstract

The complex interactions between genes and the environment play important roles in disease susceptibility and progression. One of the chronic diseases that is affected by this gene-environment interplay is cancer. However, our knowledge about these environmental factors remains limited. The microorganisms that inhabit our bodies have recently been acknowledged to play a crucial role as an environmental factor, to which we are constantly exposed. Studies have revealed significant differences in the relative abundance of certain microbes in cancer cases compared with controls. It has been reported that changes in the composition of normal gut microbiota can increase/decrease cancer susceptibility and progression by diverse mechanisms including, but not limited to, inflammation—a well-known hallmark of carcinogenesis. The microbiota can also affect the response to various treatments including immunotherapy. The microbiome-immune-cancer axis will continue to provide insight into the basic mechanisms of carcinogenesis. In this review, we provide a brief understanding of the mechanisms by which microbiota affects cancer development, progression, and treatment.

## 1. Introduction

The number of microbial cells in the human body was initially thought to be approximately 10-fold more than the sum of our own cells [1], suggesting the importance of their abundance in the human body. A recent study has shown that the estimation of these numbers is not true and that the ratio between the number of human and microbial cells in a human body is 1 : 1 [2]. However, this finding in no way undermines the active roles our microbiome plays in the body; on the contrary, it signifies that regardless of the ratio of microbial cells to human cells, the microbiome is capable of contributing to the physiological processes. Based on next-generation sequencing platforms [3, 4], it is known that the composition of microbial communities varies across different anatomical sites [5, 6]. Most microbes are bacteria, viruses, and fungi residing within our gastrointestinal (GI) tract. These together make up the human microbiome (bacteriome, virome, and fungome). However, there are differences in the microbiome composition between species and within the same species [6, 7], mainly attributed to host genetics and environmental factors, and their interactions with each other. Human disease susceptibility is primarily influenced by gene-environment interactions, and the microbiome is now believed to be a critical factor. Differences in the microbiome are evident between cases and controls for a growing list of human diseases including Crohn's disease, type-2 diabetes, autism, and chronic allergies [5, 8, 9]. In the past decade, studies have indicated that disturbance in the composition of normal microbiota influences cancer development and progression, as well as response to therapy.

## 2. Role of Microbiota in Cancer

Microbiota composition varies with tissues, indicating that their effects on inflammation and carcinogenesis are tissue-specific. The interindividual variability of microbiomes [10] determines key differences in disease development and progression. There are evidences of tumor-promoting effects of certain microbes in spontaneous, genetically driven and carcinogen-induced cancers in different organs of germ-free animals, for example, the skin, colon, liver, breast, and lungs [11–23]. In mice, depletion of intestinal microbiota using antibiotics reduces the development of cancer in the liver and colon [11, 23–30]. Although most of the studies show tumor-promoting effects of the microbiota, antitumor effects of exogenous bacterial infections have also been observed. Towards the end of the nineteenth century, antitumor effects were observed in patients with sarcomas, after bacterial infections which was later developed as Coley's toxin (heat-inactivated *Streptococcus pyogenes* and *Serratia marcescens*). Similarly, for over 40 years, one of the standard treatments for bladder cancer is BCG (mixture of bacterial extracts from *Bacillus Calmette–Guérin*) [31]. Later studies showed that specific bacterial components, such as Toll-like receptor (TLR) and NOD-like receptor (NLR) agonists, were responsible for many antitumor effects. This led to the concept that activation of innate immunity may convert tumor tolerance into antitumor immune responses [30, 32–34]. Microbes are recognized by multiple pattern recognition receptors (PRRs), which monitor the microbial status and barrier integrity, and initiate regulatory responses. These PRRs not only may control the microbiota through antibacterial mediators and thereby suppress cancer but also may promote resistance to cell death and trigger cancer-promoting inflammation. Moreover, the microbes release carcinogenic molecules, such as genotoxins and tumor-promoting metabolites [35]. The recognition of microbial patterns by TLRs is a powerful proinflammatory stimulus and a major effector of innate immunity [36]. It is well established that microbe-associated molecular patterns (MAMPs) and TLRs promote carcinogenesis. TLR4, the receptor for Gram-negative bacterial cell wall component LPS, promotes carcinogenesis in the liver, pancreas, colon, and skin, as shown by reduction in tumor development in *Tlr4*-deficient mice [37–40], and increases tumor load in mice that express constitutively activated components like peptidoglycan and lipoteichoic acid, promoting gastric cancer [41]. A key cancer-promoting downstream action of TLR signalling involves induction of survival pathways by activation of nuclear factor-*κ*B (NF-*κ*B) and STAT3 [17, 34, 39].

The composition and role of the human virome in health are understudied. A completely new avenue of research involving the viruses inhabiting the human body has changed the way viruses were looked upon. A phage is a virus that is known to infect only prokaryotic cells and not interact with eukaryotic cells. The human body has an abundance of these bacteriophages, mainly populating the areas of the blood, lymph, and organs. However, the mechanisms employed by the phages to cross epithelial barriers and access the body's organs have not yet been identified. A recent study reported that there was apical-to-basal transcytosis with every type of phage investigated across different cell lines. However, paracellular transport across an intact epithelial barrier was not found to be a likely mechanism of transcytosis [42]. This study also revealed that phages have access to membrane-bound vesicles and the cytosol. Further investigation showed that bacteriophages were found in all subcellular fractions of the eukaryotic cell with intracellular transport probably trafficking through the Golgi apparatus [42].

The main reservoir of phages in the human body is the GI tract. These phages have coevolved with the gut bacteria over the course of our life, and they have the potential to prevent pathogenic attack to their host. The presence of phages throughout the human body is very well documented. Unfortunately, articles on the issue of the microbiome in health and disease, as well as the role of microbial interactions with the immune system and with the intestinal mucosa, hardly explain the role of phages [43]. Phages, however, have been found to have antitumor effects in mouse models of melanoma [44].

As mentioned earlier, the microbiome also consists of a huge number of fungi which has been collectively named as mycobiome or fungome [45]. Despite the potential significance of the mycobiome, only few studies have analysed its composition. Great interindividual variation in mycobiome and predisposition to opportunistic infections owing to this variation has been proposed by many studies [46]. Many fungal species including *Candida*, *Aspergillus*, and *Cryptococcus* have been found to inhabit and influence infections in the human body [46]. There are studies suggesting an antagonistic relationship between *Pichia* and *Candida* species by different mechanisms [47]. Moreover, a negative correlation between *Candida* and *Campylobacter* in HIV-infected patients was also reported in this study, whereas in healthy subjects, no correlation between *Candida* and bacterial species was found [47]. Candida species is a well-known oral fungal pathogen, and studies have shown that infection with this species can significantly increase overall and some individual cancer risks, for example, head and neck, pancreatic, skin, and thyroid cancers [48]. A study in colorectal cancer patients has revealed dysbiosis in mycobiome characterised by change in fungal composition and ecology, which suggests the important role of gut mycobiome also in CRC [49].

Several reports with mouse models provide data on the fact that the composition of the gut microbiota is modulated by diet [50]. The composition of the microbiota differs among individuals living in different geographic regions and on the long-term diet [50]. A balanced microbial composition could be achieved through symbiosis that occurs through the consumption of balanced diets [50]. Dysbiosis, caused by an imbalanced diet, disturbs the microbe-immune interaction making the host susceptible to inflammation and diseases [50]. However, there is still a lack of understanding of how microbiome composition is modulated by diet [50].

## 3. Host-Microbiota Interaction

A key factor to develop symbiosis between host and microbes is the anatomical separation of microbial entities from the host compartments by layers of well-maintained physical barriers. Disturbance in these barriers leads to inflammation and diseases, including cancer [37]. The barriers include an intact epithelial lining that acts as a sensing system to detect and eliminate invading bacteria, the mucous layer surrounding the gut, and the low pH in the skin and stomach. Moreover, bacterial numbers and location are monitored by specific cell types: such as in the gut, Paneth cells defend the immune system by secreting antimicrobial molecules into the lumen, goblet cells secrete mucin to lubricate the intestinal contents and protect the epithelium, and in the skin, keratinocytes regulate the microbes by secreting antibacterial peptides [51, 52]. In the gut, secreted immunoglobulin A (IgA) provides additional protection against microbes and limits the access of intestinal antigens to the circulation and invasion of potentially dangerous bacterial species [53]. The gastrointestinal (GI) tract is considered the largest immunological organ in the body playing a significant role in regulating immune homeostasis. The interplay between epithelial cells, immune cells, and microbiome influences immune system mediators and thus affects the intestinal barrier [54]. The lining of the lower intestine contains finger-like projections that form structures called villi which increase the mucosal surface. Underlying the epithelium, the lamina propria contains the important antigen-presenting dendritic cells, which regulate humoral and cellular immunity [54]. Tight junctions, or the zonula occludens, interact with different proteins with their intracellular domains and regulate vesicular import and export [55]. They facilitate the passage of small ions and water-soluble molecules through the paracellular space and prevent the passage of antigens, microorganisms, and their toxins [55]. Apart from the host control mechanisms, the natural host microbiome nurtures a functional luminal barrier [56] by maintaining epithelial cell turnover and producing mucins, as well as by competing for resources, which suppresses the growth of pathogenic microbes. A classic example of the protective role of commensal microbiota is opportunistic infection with *Clostridium difficile,* which only causes disease when the normal resident gut microbiota is suppressed by antibiotics. This infection can be cured by transplantation of microbiota from healthy individuals [57]. Similarly, germ-free mice have an increased susceptibility to infection with pathogens [58]. Production of bacteriocins is another way by which the natural microbiota restricts the growth of pathogenic microbes [59]. Failures of these control mechanisms—that is, defective barrier, immune suppression, and dysbiosis—have been associated with microbe-driven carcinogenesis. These regulatory mechanisms are inextricably linked, and failure of one typically disturbs the overall equilibrium. For instance, infection with *H. pylori* not only injures host cells but also alters the gastric environment and barrier, which increases inflammation and disturbs the microbiota [60].

## 4. Microbiome in Immunoregulation

Microbiota shapes the innate and adaptive immunity significantly, although the intricate details are still unknown [61]. The development of the microbial flora at birth influences the maturation of the immune system and development of tolerance and containment of microbial infections [62, 63]. It continues throughout life via signalling through receptors of the innate immune cells, through sampling of the microbiota by adaptive immune response, and by generating metabolic products [64, 65]. For example, data from germ-free and antibiotic-treated mice show a markedly reduced response to CpG stimulation in the setting of cancer immunotherapy [66]. Upregulation of TLRs by LPS and other microbial products can activate the NF-*κ*B, c-Jun/JNK, and JAK/STAT3 pathways, which play roles in cell proliferation and immunosuppression [67, 68]. Overall, antibiotics, particularly during immunosuppression, may interfere with effective anticancer immune responses [69].

Apart from bacteria, the presence of bacteriophages in huge numbers in the human body naturally triggers the question of whether these are mere spectators of the whole interaction between the bacterial species and the immune system. The potential role of phages present in the GI tract is of special interest. Studies have reported that these intestinal phages may have immunosuppressive properties when administered *in vivo*, inhibiting both humoral and cell-mediated immunities [70, 71]. Therefore, intestinal phages not only may help eliminate harmful bacteria and reduce the number of commensal bacterial species, thus reducing the heavy bacterial load on local mucus membrane, but also may suppress local immune reactions [43], for example, inhibition of dendritic cells and NF-*κ*B [43]. This suppression plays a crucial role in maintaining immune homeostasis. Therefore, phages appear to have a protective role in the development of gut inflammation in healthy people, and any disturbance in the phage composition breaks the phage-mediated tolerance [43]. This breakdown may promote the development of inflammatory bowel diseases and other opportunistic infections [43].

## 5. Microbiota in Modulating Immunotherapy

**Figure 1:**
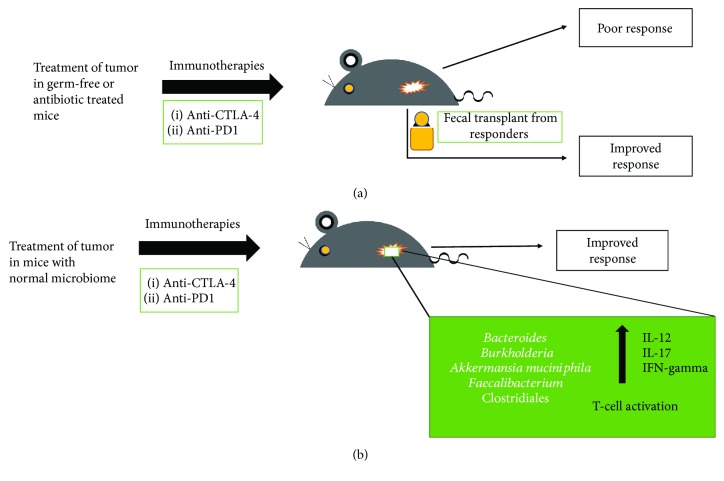
(a) Treatment of tumor in germ-free or antibiotic-treated mice shows poor response to immune checkpoint blockers. When fecal transplant is made to these germ-free or antibiotic-treated mice from responders, the mice show improved response to the same immune checkpoint blockers. (b) Treatment of tumor in mice with normal microbiome shows improved response to immune checkpoint blockers, and the prevalent species of microbiota include *Bacteroides* [74], *Burkholderia* [74], *Akkermansia muciniphila* [75], *Faecalibacterium* [77], and Clostridiales [77].

Cyclophosphamide (an immunostimulatory alkylating agent used to treat solid sarcomas) alters natural microbiota in the small intestine of mice and causes the translocation of certain gram-positive bacteria, mainly *Lactobacillus johnsonii* and *Enterococcus hirae*, into secondary lymphoid organs [72]. These bacteria stimulate the generation of a specific subset of Th17 and Th1 cells (which produce IL-17 and IFN-*γ*), underscoring how particular microbial components present in the gut lumen can regulate the polarity of Th responses to cyclophosphamide treatment. Furthermore, alteration of the gut microbiota influences the efficacy of immune checkpoint blockers (ICB). Immunotherapy has been among the most recent developments in cancer care, especially with the advent of ICBs. These inhibitors function by reactivating T cells that have been rendered ineffective by the tumor microenvironment, thus making them respond again to tumor antigens [37]. As of now, blockade of two checkpoints by monoclonal antibodies has been successful: cytotoxic T lymphocyte-associated protein 4 (CTLA-4) and programmed-cell-death protein 1 (PD-1)/programmed-cell-death ligand 1 (PD-L1) [8]. Recent research shows that the immunostimulatory and antitumor effects of the CTLA-4 antibody depend on distinct bacterial species of the gut [73]. The anti-CTLA-4 monoclonal antibody has been found to lose its therapeutic efficacy against established sarcomas, melanomas, and colon cancers in germ-free or antibiotic-treated mice (Figure 1).

A seminal study reported that response to an ICB could be improved by changing the gut microbiome of a mouse [74]. Data for many patients with different types of cancer were examined. Some of these patients were on antibiotic therapy for routine causes like dental pain or a urinary tract infection before or shortly after starting a PD-1 drug. It was found that certain bacteria of the genera *Bacteroides* and *Burkholderia* were responsible for the antitumor effect of the microbiome [74] (Figure 1). Interleukin-12 is released in response to these *bacterial species*, which may aid in triggering immune responses by stimulating the T cells [74]. To confirm the results, microbes were transferred into mice that had no intestinal bacteria, either by feeding them with the microorganisms or by giving them the *Bacteroides*-rich feces of some ipilimumab-treated patients. In both cases, growth of these bacterial species improved the response to a checkpoint inhibitor [74]. Later, studies on the differences in the gut bacteria of responders and nonresponders revealed the presence of *Akkermansia muciniphila*, a bacterial species associated with mucus lining of the gut that may provide protection against obesity and diabetes. Germ-free mice devoid of gut bacteria responded better to PD-1 blockers on receiving fecal transplants from responders, compared to mice receiving feces from nonresponders. On feeding them *A. muciniphila*, *p*oorly responding mice could be turned into responders [75].

Studies have also found that differences in composition of gut microbiota could explain why mice purchased from different vendors showed different responses to PD-1 blockers [76] (Figure 1). In a recent study, it was reported that the gut microbiome significantly affects melanoma patients receiving PD-1 blockers [77]. Like other studies, mice that received fecal transplants from responders showed better response to drugs compared to the mice that received fecal transplants from nonresponders. In this report, the bacterial species found were mainly *Faecalibacterium* and Clostridiales [77] (Figure 1).

## 6. Concluding Remarks

The crosstalk between the natural host microbiome and immune system clearly modulates local and systemic inflammatory responses, oncogenic signalling, and tumor progression. The microbiome-induced innate and adaptive immune responses have an impact on the efficacy of immunotherapy. It is therefore imperative to uncover the underlying immune mechanisms and find targetable molecules associated with the host's personal microbiota that influence immune responses. It has been shown that transplants of certain microbes restore eubiosis in chronic disease states, which reduces inflammation induced by microbial dysbiosis. Narrow-spectrum and nonabsorbable antibiotics may be used to target genotoxic or translocating bacteria. Since host diet affects normal microbiota, natural restoration of commensals through foods that help them thrive could reduce the harmful effects of chronic diseases. Genetically manipulated species of microbiota expressing or lacking specific enzymes [73] along with matched diets might be used to achieve higher levels of tumor-suppressive effects or lower levels of tumor-promoting effects or suppress the growth of tumor-promoting bacterial species [37]. Targeting the inflammatory pathways that are activated by the translocated bacterial species may reduce inflammation and slow down tumor growth and/or enhance the efficacy of certain immunotherapy strategies.

Targeting bacterial genotoxins and enzymes that promote cancer could be useful. Understanding the multifarious mechanisms by which microbiota promotes carcinogenesis will open new avenues to identifying diagnostic, preventative, and therapeutic approaches. Continued unravelling of natural microbiota and its alteration during infections, antibiotic therapy, and varied diets could lead to identification of biomarkers that determine the escape phase of an abnormal cell from immunological pressures. Intratumor heterogeneity and response to therapy can also be explained based on differences in microbial composition. Therefore, it is possible that combining anticancer therapy with certain microbes known to provide protection from cancer may be considered in the future. Certain microbial peptides have anticancer effects. For instance, azurin, which is secreted by *Pseudomonas aeruginosa*, has been found to work well against tumors [78]. Therefore, biochemical analysis of microbial peptides with potential anticancer activities could be helpful. Further insight into the microbiome-immune interplay may aid in the development of preventative vaccines against cancer. Culture conditions supporting growth of most microbes inhabiting the human body, especially anaerobic bacteria residing deep within our GI tract, need to be established. These studies should be combined with epidemiological data, genome-wide association studies, and metabolomics. It is necessary to culture specific bacteria to analyse their functional role in gnotobiotic mouse models in which either the microorganisms are excluded or their composition is known. Improved probiotic/prebiotic strategies to prevent diseases may be developed. Immunotherapy might be improved based on the knowledge of microorganisms that influence their efficacy. Since microbiota varies in different tissues, it could provide information about factors that cause certain cancers to be more aggressive. Microbiome signatures in different cancers could be developed for research on personalized medicine.
